# Is food produced by farmers healthier, more natural, and gaining more popularity? Research on the influencing mechanism of food producer labels on consumers’ food choices

**DOI:** 10.3389/fpubh.2023.1255023

**Published:** 2023-10-19

**Authors:** Yong Zhu, Xiaotong Jin

**Affiliations:** ^1^School of Business and Management, Jilin University, Changchun, Jilin, China; ^2^School of Management and Economics, Chuxiong Normal University, Chuxiong, Yunnan, China

**Keywords:** food choice, food producer label, perceived naturalness, standardization perception, food processing level

## Abstract

**Introduction:**

Extant studies have demonstrated the relationship between naturalness and healthiness, and the effectiveness of various food labels in influencing consumers’ perception of food and subsequent food choices. However, little attention has been given to food producer labels.

**Methods:**

Drawing on Stimulation-Organism-Response theory, the current study explored the causal relationship between food producer labels and consumers’ food choices. Three studies (562 participants) were employed to test the main effect, the mediating effect, and the moderating effect.

**Results:**

The results showed that: (1) food producer label could influence consumers’ food choice, that is, produced-by-farmer label (vs. produced-by-enterprise label vs. control group) could significantly increase consumers’ food choices, while there is no significant difference between produced-by-enterprise label and control group. (2) Perceived naturalness and standardization perception mediate the effects on consumers’ food choices of food producer labels. (3) Food processing level moderates the effect of food producer label on consumers’ food choices.

**Discussion:**

The current study enriches the research of food label and food choice, expands the application of Stimulation-Organism-Response theory in consumer behavior, and provides some practical suggestions for consumers, enterprise and policy-maker. Various kinds of experiments (online and offline) enhanced the conclusions’ ecological validity. Finally, the limitations and future research are discussed.

## 1. Introduction

Food is a fundamental part of our daily lives. A food label provides some significant information to consumers, and it is widely used in marketing, e.g., clean food label (1, 2), genetically modified food label (3–5), organic label [(6); B (7)], hygiene warning label (8), all natural label (9), Eco-label (10, 11), fair trade label [(12); Gunne (13)], traffic-light label (14, 15), guideline daily amount label (16), menu label (17), expiration date label (18), etc. and such food labels, have been proven to be effective in influencing consumers’ food purchasing intention and consuming behaviors. However, very few studies have examined the food producer label. It is possible that consumers may have different perceptions of different food producers (19), which means that they might judge the quality of food based on that perception. In the context of food production by individuals and enterprises, the unexplored food producer label (produced-by-farmer vs. produced-by-enterprise vs. control group) seems to be significant and worthy of further investigation.

## 2. Literature review and hypotheses development

### 2.1. Food labels and food consumption

A small food label can have a significant impact on food choices (20). ***Food label*** is an important communication tool that provides consumers with information about a product’s composition, nutritional profile, and quantity of contents so that they can make product comparisons and selections (21). ***Food choice*** is the process by which individuals and households decide what and how to produce, acquire, prepare, distribute, and consume food (22). Obviously, ordinary citizens do not need to produce and distribute the food all by themselves. However, most of ordinary citizens need to buy, eat, or even recommend food in their daily life. Hence, *the current study defines food choice as the process by which individuals and households decide what to buy and eat food, and whether to recommend the food*. Previous researches have studied some specific food labels’ effects on food choices and consumption, i.e., clean food label, GMO label, organic label, hygiene warning label, all natural label, Eco-label, fair trade label, traffic-light label, GDA label, menu label, shelf label, etc. Specifically, some researchers empirically find that organic label significantly increases the health perceptions of a product, i.e., consumers generally perceive organic labeled foods to be healthier than conventional foods (G (23).). Meanwhile, the presence of an organic label can lead to a bias in consumer perception of the product, often referred to as the halo effect which has given some food companies the opportunity to use the organic label to attract health-oriented customers by adding organic labels to non-organic foods (24). In addition to influencing consumers’ perceptions of health, organic labels further improve their perceptions of safety. Food processing safety and hygiene warning labels can significantly reduce consumers’ perceptions of risk (8). Furthermore, food label also raises moral perceptions among consumers, with GM food label more often triggering negative moral perceptions and reducing consumers’ willingness to pay, as consumers generally perceive GM technology as an unethical manipulation of the laws of nature (25), while the presence of an Eco-label increases consumers’ moral perceptions of the retailer. On the one hand, this is because eco-friendly products themselves demonstrate a sense of corporate social responsibility. On the other hand, it also significantly increases the social identity of individuals who purchase such products (26). Thus, food labels significantly affect consumers’ food perceptions, including the impact on consumers’ perceptions of health, the perceptions of safety and risk, and the perceptions of morality. On the basis of the above results, existing literatures demonstrate that food labels further influence consumers’ trust in products (27), willingness to pay and purchase intentions (28, 29), and food choices.

Food labels serve as a primary source of information for consumers about food, and they have a significant impact on consumers’ food choices (30). In this case, consumers are looking for food information to judge the characteristics and attributes of the food, and food labels can enable them to discern pertinent information. Farmers earn income from planting and selling produce (31, 32), which are the original ingredients of most food. People, therefore, always make an implicit connection between farmers and food, especially the produce. We could thus conclude that consumers might prefer to buy and consume food if they find some information about farmers (vs. enterprise vs. control group) on the surface or package of the food. Meanwhile, the company employs large-scale and standardization operations, and when customers discover the corporate message on food products’ surface or packaging, they may feel assured of food quality. The enforcement of laws and the effectiveness of regulatory institutions also make customers subconsciously believe that the quality of food is guaranteed, even if there is no information on the food products’ surface or packaging. Hence, we infer that there lies no difference of the effect between produced-by-enterprise label and the control group. The **Simulation-Organism-Response theory** (SOR theory) assumes that the environment contains stimuli (S) that cause changes to people’s internal or organismic states (O), which in turn cause approach or avoidance responses (R) (33). Based on SOR theory, produced-by-farmer label (vs. produced-by-enterprise vs. control group) is a stimulation (S) that could increase individuals’ perceived naturalness (O), which further enhances consumers’ food choices (R). Thus, we propose the following hypothesis. Formally stated:

*H1:* Food producer label (produced-by-farmer label vs. produced-by-enterprise label vs. control group) could influence consumers’ food choice. Specifically, consumers show a higher willingness to purchase food when they are faced with produced-by-farmer label (vs. produced-by-enterprise label vs. control group), and there is no significant difference between produced-by-enterprise label and control group.

### 2.2. Perceived naturalness and standardization perception

Humans have been in natural environments for most of their evolutionary life, and nature has a positive effect on physical and mental health, cognitive functioning, curriculum learning, increasing levels of well-being and positive thinking, and reducing aggression and violence (34). Farmers have a higher implicit association with nature compared with enterprises, thus farmer-related information gives consumers a deeper sense of perceived naturalness than enterprise-related information. ***Perceived naturalness*** is the degree to which consumers perceive a product to be natural in terms of appearance, workmanship and other dimensions (35), and the natural property of an object is one of the key indicators of its quality for consumers (L. (36)), because people have the “naturalness equals healthiness” bias (37), and consequently prefer naturalness, especially for food, even the natural and artificial objects are specified as equivalent (38). The biophilia hypothesis proposes that humans have evolved over time to genetically prefer certain natural environments that help them have an increased chance of survival, escaping from danger and access to food (39). The stress recovery theory suggests that three elements, i.e., non-threatening landscape elements, greenery elements, and specific natural landscapes, are effective in reducing stress and stimulating positive emotions in humans (40). All these studies suggest that perceived naturalness increases positive human emotions. The presence of a farmer-owned label improves consumers’ food choices, as has been empirically demonstrated using Dutch consumer milk consumption data, which shows that not only does the farmer-owned label increase consumers’ willingness to buy, but also that consumers are willing to pay a premium for milk containing the farmer-owned label (41). Based on SOR theory (33), produced-by-farmer label is a stimulation (S) that could increase individuals’ perceived naturalness (O), which further enhances consumers’ food choices (R).

The commodity flow path shows that agricultural products are processed into food and traded on markets, and that food standardization increases the perceived quality reliability of the commodity. ***Food standardization*** means that the production, processing and distribution of food are done according to specific standards. A standard is a set of details and criteria that must be adhered to throughout the whole process in order to be successful. From food production and processing to sales, each process requires scientific and technological attention and management (42). There is evidence that marketing standardization (43) and channel management standardization (44) are significantly associated with firm performance. Standardization significantly contributes to firm performance in the production industry and not so significantly in the service industry (45). However, the effect of standardization on service quality exceeds the effect of customization on service quality (46). As can be seen from the above, the majority of studies on standardization in the literature have focused on enterprises, and little literature has begun to explore the feasibility of standardizing production for individual farmers, suggesting that the link between standardization and enterprise is in line with people’s common sense. According to SOR theory (33), when consumers are confronted with a produced-by-enterprise label (vs. produced-by-farmer label), it activates the consumer’s ***standardization perception***. On the contrary, produced-by-farmer label (vs. produced-by-enterprise label) could decrease consumers’ standardization perception, consequently weakening consumers’ food choice. Additionally, most of foods’ ingredients are from produce, and people always make an implicit connection between farmers and the produce. Therefore, produced-by-farmer label (vs. produced-by-enterprise label) could increase consumers’ perceived naturalness and decrease standardization perception, and the effect on perceived naturalness is bigger than that of standardization perception. Thus, we develop the following hypothesis. Formally stated:

*H2:* Perceived naturalness and standardization perception parallel mediate the effect of food producer label on consumers’ food choice. Specifically, perceived naturalness positively mediates the main effect, while standardization perception negatively mediates the main effect, and the effect of perceived naturalness is bigger than that of standardization perception.

### 2.3. Food processing level

***Food processing*** has increased the diversity of nutritious foods in the modern diet (47) and has met the needs of consumers for food diversity. Depending on the degree of processing and the purpose of processing, food products can be classified into different types. Among these classifications, the **NOVA *classification*** of processed foods is popular and widely accepted (48, 49), and scholars’ studies on food classification have mostly adopted the NOVA classification or expanded their exploration based on the original NOVA classification (50, 51). NOVA divides food into four categories. Firstly, raw or minimally processed foods, such as fruit, rice, etc. Secondly, culinary ingredients, raw foods that have been refined for cooking, such as edible oil made from nuts, rape flowers, etc. Thirdly, processed foods, foods that have been processed with salt, oil, sugar, etc. Their properties have been altered as a result of these processing, e.g., canned fish, fresh bread, cheese, etc. The fourth category is ultra-processed foods, i.e., foods that are processed on the basis of processed foods, which usually contain more than or equal to five industrial ingredients and are high in sugar, fat and calories, such as sugary drinks, biscuits, ham sausages, ice cream, cakes, etc. (52, 53). It is very clear that the more processed the food is and the more food additives it has, the less healthy it is, and processed and over-processed foods are often considered to be of low nutritional quality and unhealthy (54). The unnatural and unhealthy properties of ultra-processed foods are particularly pronounced (55). In conclusion, the properties of 3^rd^ and 4^th^ food are altered by processing, while the properties of 1^st^ and 2^nd^ food are not (52, 53, 56), which could influence perceived naturalness, standardization perception, and consumers’ food choice. Specifically, for 1^st^ (raw or minimally processed foods) or 2^nd^ (culinary ingredients) foods, the properties of foods keep unchanged, and produced-by-famer label (vs. produced-by-enterprise label) is a stimulation (S) that could increase individuals’ perceived naturalness and decreases standardization perception (O), which further significantly enhances consumers’ food choices (R). For 3^rd^ (processed foods) or 4^th^ (ultra-processed foods) foods, the properties of foods (especially some properties about naturalness) have been alerted, there is no statistically significant difference in the effects of food producer labels (produced-by-farmer vs. produced-by-enterprise) on consumers’ food choices. Therefore, we develop the following hypothesis. Formally stated:

*H3:* Food processing level plays a moderating role in the effect of food producer label on food choices. Specifically, for 1^st^ or 2^nd^ foods, produced-by-farmer label (vs. produced-by-enterprise label) could increase their food choices. For 3^rd^ or 4^th^ food, there is no statistically significant difference in the effects of food producer labels (produced-by-farmer vs. produced-by-enterprise) on consumers’ food choices.

## 3. Study design, experiments, and study results

We tested our hypotheses with 3 studies. Study 1 verified the causal relationship between food producer label and food choices with 2 kinds of foods, that is, consumers showed a higher willingness of food choices when they faced produced-by-farmer label (vs. produced-by-enterprise label vs. control group), and there was no significant difference between produced-by-enterprise label and control group. Study 2 tested the parallel mediating effects of perceived naturalness and standardization perception of food producer labels on consumers’ food choices. Specifically, perceived naturalness mediated the effects of produced-by-farmer labels on food choices, and standardization mediated produced-by-enterprise labels’ impact on food choices. Study 3 explored the boundary condition of food processing level on the effect of food producer labels on consumers’ food choice.

### 3.1. Study 1: test of the main effect

#### 3.1.1. Design, participants, and procedure

Study 1 used a between-subjects design experiment with three manipulated condition (food producer label: produced-by-farmer label vs. produced-by-enterprise label vs. control group). After an estimation of the sample size with G-Power, 160 Chinese residents (65.00% females; Mage = 29.54, SD = 7.90) were recruited from an online experiment platform named CREDAMO for monetary reward, and 5 participants were excluded from the study for their answers being beyond 3 times of standard deviation, remaining a final sample of 155 (*n_produced-by-farmer_* = 54, *n_produced-by-enterprise_* = 48, *n_control_* = 53). After the participants agreed to participate in a questionnaire study about The Willingness of Orange Choices with 3 items, they were told that:

Imagine one day you were wandering in the food market and you came across a fruit stall and saw oranges as shown in the picture (see Supplementary Figure 1). How would you make the following decision? (1) How likely are you to buy the oranges? (2) How likely are you to eat the oranges? (3) How likely are you to recommend the orange to your relatives or friends? (1 = not at all likely, 7 = very likely; α = 0.837).

#### 3.1.2. Results and discussion

The three food choices measuring items were averaged to create an index. In support of hypothesis 1, a one-way ANOVA revealed a significant main effect of food producer label on consumers’ food choices [*F*(2,152) = 9.803, *p* = 0.000, η^2^ = 0.114]. Specifically, the participants of produced-by-farmer label (M = 5.963, SD = 0.723) reported higher likely of food choice than control group [M = 5.208, SD = 0.752, *t*(105) = 5.299, *p* = 0.000, Cohen’s d = 1.024] and those of produced-by-enterprise label [M = 5.396, SD = 1.227, *t*(100) = 2.880, *p* = 0.005, Cohen’s d = 0.563]. Additionally, there was no significant difference between the produced-by-enterprise and the control group [*t*(99) = 0.99, *p* = 0.350]. All the results supported hypothesis 1.

#### 3.1.3. Supplementary study

To verify the robustness of the results of hypothesis 1, the participants (*N* = 160, 65% female, M_age_ = 29.54, SD = 7.90) who had participated in the above study, were instructed to fill out another survey named The Willingness of Meat Choices with 3 items. All 160 participants were kept for the study (*n_produced-by-farmer_* = 54, *n_produced-by-enterprise_* = 53, *n_control_* = 53). They were told that, someday, you went to a supermarket for shopping and wandered into the meat section and saw pork as shown in the picture (see Supplementary Figure 2), what your choices were for the following questions: (1) How likely you are to buy the pork? (2) How likely you are to eat the pork after cooking as you wish? (3) How likely you are to recommend the pork to your relatives or friends? (1 = not at all likely, 7 = very likely; α = 0.894). Being identical with the above study, the supplementary study supported hypothesis 1. A one-way ANOVA showed the significant effect of food producer labels on consumers’ food choice [*F*(2,157) = 6.534, *p* = 0.002, η^2^ = 0.077]. The participants of produced-by-farmer label (M = 5.691, SD = 0.878) reported higher likely of food choice than control group [M = 5.239, SD = 1.301, *t*(105) = 2.111, *p* = 0.037, Cohen’s d = 0.407] and those of produced-by-enterprise label [M = 4.748, SD = 1.737, *t*(105) = 26.62, *p* = 0.001, Cohen’s d = 0.685]. Additionally, there was no significant difference between the produced-by-enterprise group and the control group [*t*(104) = 1.645, *p* = 0.103]. Hypothesis 1 was tested again by the study 1B.

Study 1 proved the main effect, as we expected, that food producer labels could influence consumers’ food choices, that is, the participants from produced-by-farmer label group reported higher levels of food choices than control group and those of produced-by-enterprise label group, and there was no significant difference between the produced-by-enterprise group and control group (Hypothesis 1). With supplementary study, we validated hypothesis 1 in another context and with a different type of food in order to ensure the robustness of the main effect. Our following work aimed to test the mediating role of perceived naturalness and standardization perception, which could provide insights into how the main effect occurs.

### 3.2. Study 2: test of the mediation of perceived naturalness and standardization perception

#### 3.2.1. Design, participants, and procedure

As tested in study 1, there was no significant difference between produced-by-enterprise label and control group, thus study 2 used a one-factor (food producer label: produced-by-farmer label vs. produced-by-enterprise label) between-subjects design. 150 CREDAMO workers (63% female, Mage = 43.19, SD = 161.72) took part in the study for monetary payment, and one participant was removed from the study because he/she guessed the purpose of the study, leaving 149 participants for the study (*n_produced-by-farmer_* = 74, *n_produced-by-enterprise_* = 75). First of all, the participants reported their personal information about gender, age, income, purchasing experience and preference of baby Chinese cabbage. After that, they were told to participate in the investigation named ***The Willingness of Baby Chinese Cabbage Choices***, they read the following instruction: *Imagine you are wandering at a wholesale vegetable market and find baby Chinese vegetables as shown in the picture (see Supplementary Figure 3), what your choices of the baby Chinese cabbage are, consisting of 3 items, (1) How likely are you to buy the baby Chinese cabbage? (2) How likely are you to eat the baby Chinese cabbage after cooking as you wish? (3) How likely are you to recommend the baby Chinese cabbage to your relatives or friends?* (1 = not at all likely, 7 = very likely; α = 0.806). Then all the participants answered the questions of perceived naturalness, whose scale was derived from Hagen’s research (37), rating the extent to which they thought the baby Chinese cabbage was “natural” “pure” and “unprocessed” (1 = not at all, 7 = very much; α = 0.883). Along with perceived naturalness, the participants rated the degree to which the baby Chinese cabbage was “production standardization” “logistics standardization” and “sale standardization” (α = 0.817), which referred to Yuan’s study (57). Besides, they were instructed with some questions about the degree of their current emotion, i.e., fear, anxiety, sadness, happiness, on a one-item, 7-point Likert scale, respectively. Finally, they answered some questions about alternative mediating variables, i.e., flavor perception (58), and degree of involvement (59).

#### 3.2.2. Results and discussion

A one-way ANOVA was used to compare the food choice of baby Chinese cabbage between produced-by-farmer label and produced-by-enterprise label. Results showed a significant difference between conditions such that the participants of produced-by-farmer label (M = 5.856, SD = 0.702) reported higher likely of food choice than those of produced-by-enterprise label [M = 5.373, SD = 1.139, *F*(1,147) = 9.657, *p* = 0.002, η^2^ = 0.062]. We tested gender, age, income, purchasing experience and preference of baby Chinese cabbage’s effects and final results showed that gender (*p* = 0.037) and preference (*p* < 0.001) had a significant effect on consumers’ food choice, while age (*p* = 0.442), income (*p* = 0.098) and purchasing experience (note: all the participants had purchasing baby Chinese cabbage experience) did not exert a significant effect on food choice. Thus, gender and preference were regarded controlling variables in the following analysis. After controlling gender and preference, food producer labels still significantly influenced consumers’ food choices [*F*(1,145) = 8.946, *p* = 0.003, η^2^ = 0.058].

**Figure 1:**
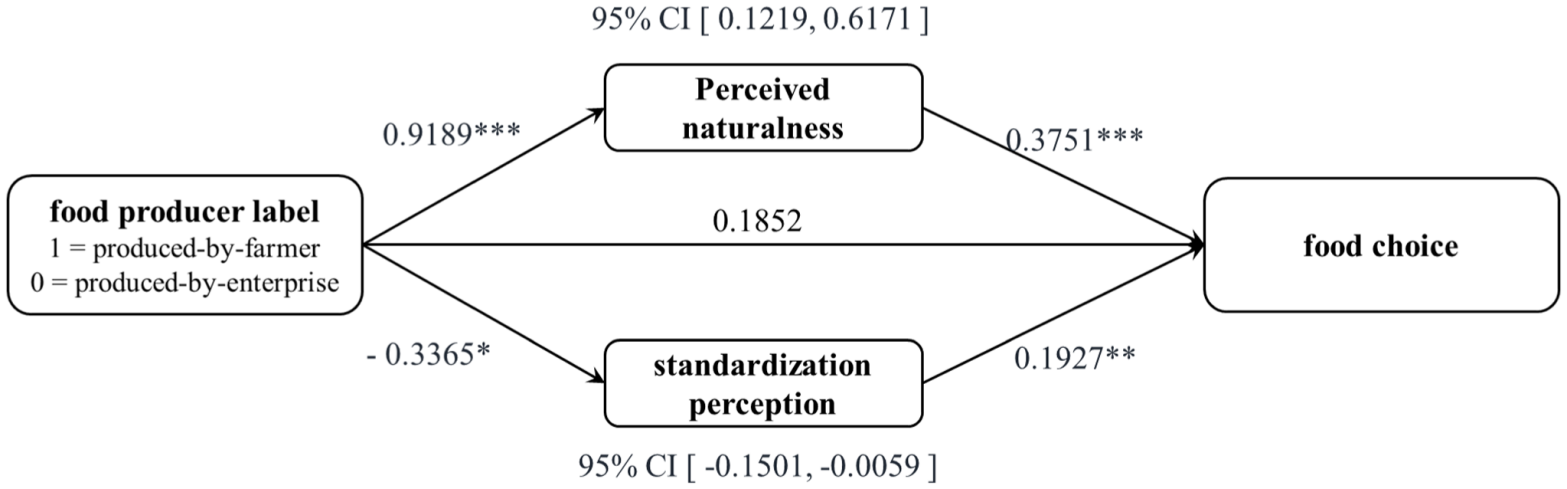
The parallel mediation model. * = *p* < 0.05; * = *p* < 0.01; * = *p* < 0.001.

An ANOVA analysis, using food producer label as independent variable (produced-by-farmer = “1”, & produced-by-enterprise = “0”) and perceived naturalness as dependent variable, revealed that produced-by-farmer label influenced consumers’ perceived naturalness much more than produced-by-enterprise label [M*_produced-by-farmer_* = 5.698, SD = 0.672; M*_produced-by-enterprise_* = 4.764, SD = 1.382; *F*(1,147) = 27.404, *p* = 0.000, η^2^ = 0.157]. In the same way, standardization perception as dependent variable revealed that the expected effect was observed [M*_produced-by-farmer_* = 5.378, SD = 1.021; M*_produced-by-enterprise_* = 5.676, SD = 0.735; *F*(1,147) = 4.166, *p* = 0.043, η^2^ = 0.028]. Next, perceived naturalness and standardization perception were used as mediators using the procedures outlined in Piters’ research (60). A bootstrapping technique with 95% confidence intervals and 5,000 samples was employed to test for mediation (model 4) (61). The results showed significant indirect effects of food producer label on consumers’ food choice through perceived naturalness, β = 0.3447, 95% CI [0.1219, 0.6171], and standardization perception β = −0.0648, 95% CI [−0.1501, −0.0059], respectively. Zero did indeed fall outside of the interval, providing statistical evidence of successful mediation (see Figure 1). Finally, we ruled out some alternative mediating variables for the main effect, such as fear [CI: −0.0645, 0.0419], anxiety [CI: −0.0370, 0.0450], sadness [CI: −0.0228, 0.0664], happiness [CI: −0.0596, 0.1592], flavor perception [CI: −0.0603, 0.3442], and involvement [CI: −0.0895, 0.2157] because all the intervals included zero (see Figure 1).

Study 2 proved the expected main effect (hypothesis 1) again and tested the rationalization of the parallel mediating model of perceived naturalness and standardization perception (hypothesis 2). In the following research, we were trying to find a good moderating variable fit for the main effect.

### 3.3. Study 3: the moderating effect of food processing level

Study 3 had several goals. Our first objective was to gain insight into how food producer labels influenced consumers’ food choices. Second, we used our study to validate the moderating effect of food processing level on consumers’ food choices from food producer labels.

#### 3.3.1. Design, participants, and procedure

After an estimation of the sample size with G-Power, 272 participants were recruited from a university of Yunnan province, China, and randomly assigned to a 2 (food producer label: produced-by-farmer vs. produced-by-enterprise) × 2 (food processing level: sliced raw fish vs. fish sauce) between-design. Twenty participants were excluded for their failure at the attention test, leaving a final sample size of 252 participants (87% female, Mage =19.89, SD = 1.219; *n_produced-by-enterprise-sliced raw fish_* = 58, *n_produced-by-enterprise-sliced raw fish_* = 68, *n_produced-by-farmer-fish sauce_* = 64, *n_produced-by-enterprise-fish sauce_* = 62). Following their agreement of participating in the survey of ***Products’ Market Acceptance from Self-Owned Shop***, all participants were asked to read the following sentence:

Imagine that a self-employed shop is proposing to launch an innovative product as shown in the picture (see Supplementary Figure 4) and is conducting market research to determine whether it will eventually sell the product. Observe the picture carefully for a period of time and answer the following questions: (1) How likely are you to buy the product? (2) How likely are you to eat the product (after cooking as you wish if it needs)? (3) How likely are you to recommend the product to your relatives or friends? (1 = not at all likely, 7 = very likely).

Participants might see either sliced raw fish (2^nd^ food: culinary ingredients) or fish sauce (3^rd^ food: processed food), each of which had a produced-by-farmer label (the produced-by-farmer group) or a produced-by-enterprise label (the produced-by-enterprise group). [Note: Sliced raw fish are kinds of a product obtained by cutting fresh fish into slices, and they are not cooked by traditional methods such as stir-frying, deep-frying or steaming. Fish sauce is a product made by stir-frying fish, ginger, garlic, peppers and other ingredients in oil (see Supplementary Figure 4). According to the classification of NOVA; (52, 53), sliced raw fish is the 2^nd^ food (culinary ingredients), and fish sauce is the 3^rd^ food (processed food). Hence, sliced raw fish and fish sauce were used as experimental materials]. After they answered the questions of food choices, they reported their gender, age, monthly income, preference of fish.

#### 3.3.2. Results and discussion

Our experiment used an item (Please recall that the green label in the top right corner of the picture is: A. produced-by-farmer, B. the produced-by-enterprise, C. other content) at the end of the experiment to conduct a manipulation check. All 252 participants answered the question correctly, indicating there is no difference among them in identifying food producer labels, suggesting a successful manipulation.

**Figure 2:**
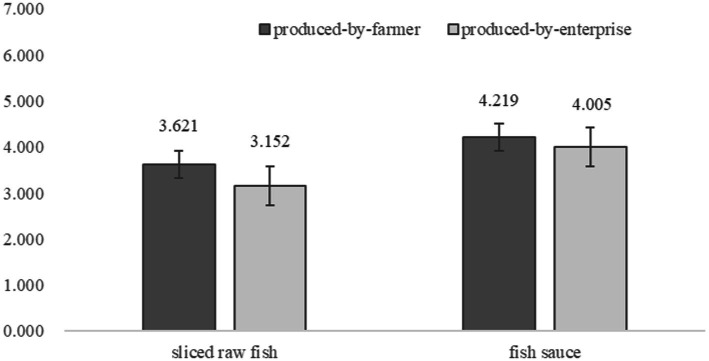
The results of moderating effect.

An ANOVA result showed that food producer labels significantly influence consumers’ food choice [M*_produced-by-farmer_* = 3.934, SD = 1.169; M*_produced-by-enterprise_* = 3.559, SD = 1.362; *F*(1, 298) = 5.478, *p* = 0.020, η^2^ = 0.021]. More importantly, the regressing result, using the interactive term of food producer label and processing level as independent variable, food choice as dependent variable, was statistically significant (*p* = 0.001). After controlling gender, age, monthly income, and preference for fish, the result kept significant too (*p* = 0.003). Specifically, for sliced raw fish, the effect of food producer labels on consumers’ food choice is statistically significant [M_produced-by-farmer_ = 3.621, SD = 1.157; M_produced-by-enterprise_ = 3.152, SD = 1.425; *F*(1,250) = 6.065, *p* = 0.015, d = 0.361]. For fish sauce, the effects of food producer labels on consumers’ food choice aren’t significant (M_produced-by-farmer_ = 4.219, SD = 1.146; M_produced-by-enterprise_ = 4.005, SD = 1.143; *p* = 0.291; see Figure 2), supporting hypothesis 3.

With different types of food and different purchasing conditions, study 3 examined the effect of food producer labels on consumers’ food choices. Most importantly, food processing level (1^st^ or 2^nd^ food vs. 3^rd^ or 4^th^ food) was found to moderate the effect of food producer label on consumers’ food choice. To this end, all hypotheses (hypotheses 1, 2, and 3) had been tested successfully.

## 4. General discussion

It is very common that many foods have labels posted on the surface of foods or on the package of foods in our daily life. These labels are to attract consumers’ attention and influence their subsequent purchasing behaviors, by conveying some attributional information about the foods. Previous researches have proved that naturalness could lead individuals perceive health, and a lot of food labels, e.g., clean food label, (non)genetically modified food label, organic label, hygiene warning label, all natural label, Eco-label, fair trade label, traffic-light label, guideline daily amount label, menu label, etc., could enhance consumers’ purchasing intention and behaviors. According to the present study, food producer labels can have a considerable impact on consumers’ food choices. Our study, to some degree, enriches the existing literature on food labels and food choices. It also provides some practical implications for business practice, consumers’ food choices and policy makers’ decision making.

### 4.1. Theoretical contributions and practical implication

The current work studies the mechanism of food producer label on consumers’ food choice, proves that food producer label (produced-by-farmer vs. produced-by-enterprise vs. control group) could affect consumers’ food choice, including purchasing, eating and recommendation, and makes several theoretical contributions to the mainstream literature on food labels and food choices. Firstly, by two different circumstances and foods, our research tests the influencing mechanism of food producer labels on consumers’ food choices, that is, produced-by-farmer label (vs. produced-by-enterprise vs. control group) could strengthen consumers’ food purchasing, food eating and food recommendation, while there is no significant difference between produced-by-enterprise and control group (study 1). Secondly, we develop a parallel mediating model to provide an explanation for the main effect (study 2). Within the parallel mediating model, perceived naturalness and standardization perception statistically significantly parallel mediate the effect of produced-by-farmer, and produced-by-enterprise, on consumers’ food choices, respectively. Finally, food processing level is tested as a boundary condition of the main effect (study 3). Specifically, for 1^st^ (raw or minimally processed foods) or 2^nd^ (culinary ingredients) foods, *produced-by-farmer label (*vs. *produced-by-enterprise label) could increase their food choices.* For 3^rd^ (processed foods) or 4^th^ (ultra-processed foods) foods, there is no statistically significant difference in the effects of food producer labels (produced-by-farmer vs. produced-by-enterprise) on consumers’ food choices. All these conclusions contribute to the current mainstream literature on food labels and food choices, and expand the application of Stimulation-Organism-Response theory in consumer behavior.

By highlighting the role of food producer labels for decision making of enterprise, consumer, and even government, our research has potentially vital practical implications. ***From a marketing perspective***, for unprocessed food, e.g., fruit, rice, flour, vegetable, (roasted) potato, tomato, cooking oil, pork, fish, etc., adding a produced-by-farmer label could enhance consumers’ perceived naturalness and consequently, increase their willingness to purchase, eat and recommend. Obviously, the effect of perceived naturalness is driven by individuals’ perceived health, which could improve consumers’ emotional value and increase their welfare. In order to increase sales, enterprises should display the produced-by-farmer label on food surfaces or food packages if the product or its original ingredients are produced by farmers. There is also the possibility that posting a produced-by-farmer label can reduce consumer food price sensitivity because people have the feeling that healthy means expensive (62). In the meantime, it is not advisable for companies to show the produced-by-enterprise label on food surfaces or packaging, since there is no statistically significant difference between the produced-by-enterprise group and the control group. Besides, posting an additional food label would increase the cost of food. However, for processed food, e.g., canned fish, fresh bread, cheese, sugary drinks, biscuits, ham sausages, ice cream, cakes etc., it is not effective to include a produced-by-farmer label because there is no significant difference between the produced-by-enterprise group and the produced-by-farmer group. Additionally, ***ordinary consumers*** should be aware that, under the principle of ensuring food quality, there is no significant difference in quality and nutrition between the food labeled produced-by-farmer label and other labels. Therefore, you should focus on food price, food quality, and your own food flavor preference. Lastly, ***policy makers*** must monitor whether businesses are legal in how they implement food labelling practices in order to avoid businesses deceiving consumers through labels. Meanwhile, if policy makers plan to push guiding consumers to a healthy diet to refrain from the illness caused by fatness, they could mandate companies to post the produced-by-farmer label on healthy foods appropriately, if the product or its original ingredients are produced by farmers, to increase consumers’ healthy food choice and consequently enhance consumers’ and the whole society’s welfare.

### 4.2. Limitation and future work

Although we have designed and implemented two different purchasing circumstances to support the main effect (study 1), one experiments to test the mediating effect (study 2), and one experiment to validate the boundary conditions of the main effect (study 3), ensuring that our conclusions are theoretically-based, tested and robust, there still might be some limitations in our study. Firstly, the current study examined the parallel mediating effect of perceived naturalness and standardization perception on the main effect. Besides, food producer labels might elicit consumers’ health perception and hygiene perception, and both of them are not discussed. Future research could try to test the two alternative mediators. Secondly, we discussed two levels of food producer labels, i.e., produced-by-farmer and produced-by-enterprise. However, there might be some other food producer types. Even for produced-by-enterprise food, the enterprises might adopt different technologies, e.g., traditional technology or eco-environmental technology. The potential future research could explore other food producer types to enrich the study. Finally, our study focuses on the consumers’ food choice, and we think such labels might be applied in other fields, such as tourist accommodation, leisure restaurant, ethnic clothing etc. Future study could examine their applications in new fields.

## Data availability statement

The raw data supporting the conclusions of this article will be made available by the authors, without undue reservation.

## Ethics statement

The studies involving humans were approved by Association for Science and Technology of Chuxiong Normal University, China. The studies were conducted in accordance with the local legislation and institutional requirements. The participants provided their written informed consent to participate in this study.

## Author contributions

XJ: Conceptualization, Writing – review & editing, Project administration. YZ: Conceptualization, Data curation, Formal Analysis, Investigation, Methodology, Software, Validation, Visualization, Writing – original draft, Writing – review & editing.
