# Supplementary material for: Is food produced by farmers healthier, more natural, and gaining more popularity? Research on the influencing mechanism of food producer labels on consumers’ food choices
Source: Front Public Health. 2023 Oct 19;11:1255023. doi: 10.3389/fpubh.2023.1255023 (PMC10622668; doi:10.3389/fpubh.2023.1255023)
Supplement: Supplementary file 1 [file Image_1.pdf]

Supplementary Material

Fig. 1 The Materials of study 1A: orange

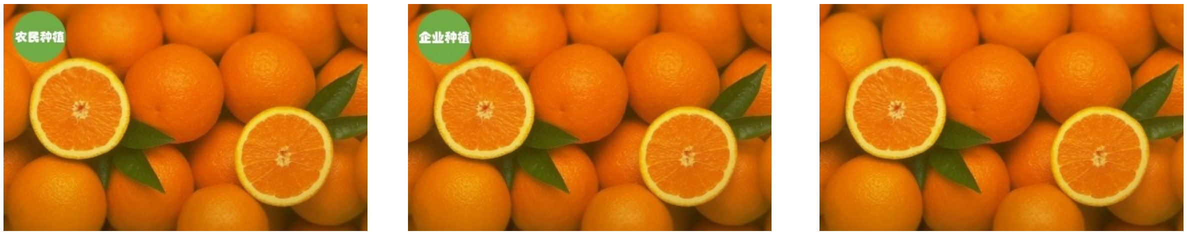

1a. produced-by-farmer group 1b. produced-by-enterprise group 1c. control group

Note: In Chinese, ‘农民种植’ means produced-by-farmer, ‘企业种植’ means produced-by-enterprise.

Fig. 2 The Materials of study 1B: pork

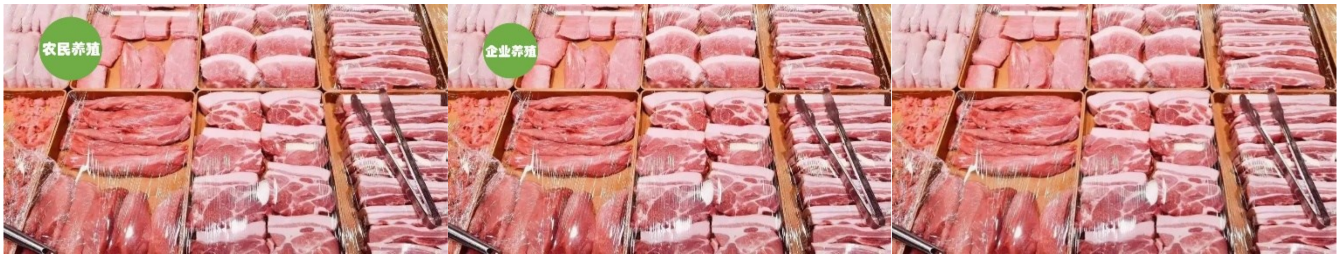

2a. produced-by-farmer group 2b. produced-by-enterprise group 2c. control group

Fig.3 The Materials of study 2: baby Chinese cabbage

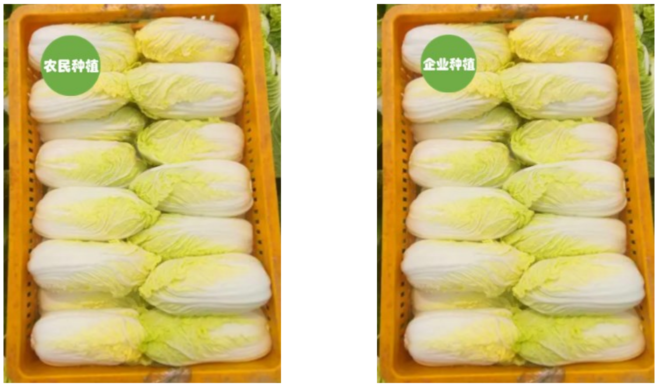

3a. produced-by-farmer group 3b. produced-by-enterprise group

Fig.4 The Material of study 3: sliced raw fish & fish sauce

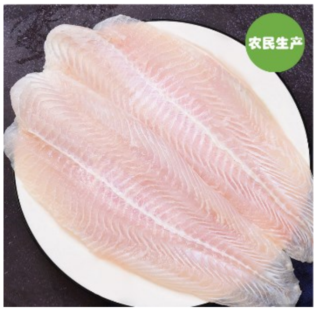

4a. produced-by-farmer group

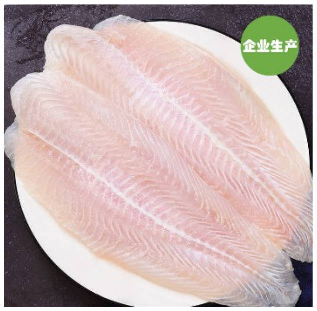

4b. produced-by-enterprise group

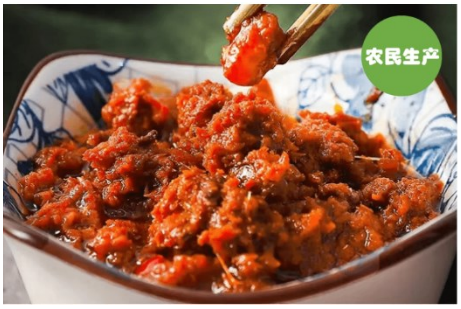

4c. produced-by-farmer group

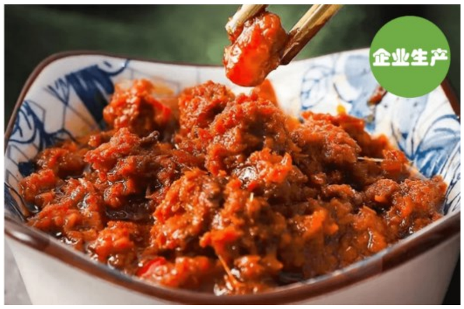

4d. produced-by-enterprise group

Note: fig. 4a & fig. 4b belong to 2nd food (culinary ingredients); 2) fig. 4c & fig. 4d belong to 3rd food (processed food).
